# Assessment of Diagnostic Values among CA-125, RMI, HE4, and ROMA for Cancer Prediction in Women with Nonfunctional Ovarian Cysts

**DOI:** 10.1155/2018/7821574

**Published:** 2018-10-08

**Authors:** Shina Oranratanaphan, Sinee Wanishpongpan, Wichai Termrungruanglert, Surang Triratanachat

**Affiliations:** Department of Obstetrics and Gynecology, Faculty of Medicine, Chulalongkorn University, Bangkok 10330, Thailand

## Abstract

**Objectives:**

To evaluate the diagnostic performance among CA-125, RMI, HE4, and ROMA for cancer detection in women with nonfunctional ovarian cysts at King Chulalongkorn Memorial Hospital (KCMH). Secondary objective is to reconsider the proper cutoff value of HE4.

**Methods:**

This is a prospective analytic study in women with nonfunctional ovarian cysts larger than 3 cm who scheduled for surgery at KCMH during 3^rd^ June 2015 to 31^st^ May 2016. Ultrasonogram and blood sample collection were completed before the operation. Patients' demographic information and pathologic results were obtained. SPSS software version 17 was used for statistical evaluation.

**Results:**

A total of 281 participants were evaluated. 19.9% of them were malignant. Compared with CA-125, HE4 had lower sensitivity (53.4% vs. 87.9%) and NPV (89% vs. 93.6%) but higher specificity (97.8% vs. 46.2%) and PPV (86.1% vs. 29.8%). ROMA had slightly lower sensitivity (79.3% vs. 87.9%) and similar NPV (93.7% vs. 93.6%), but higher specificity (79.8% vs. 46.2%) and PPV (50.5% vs. 29.8%) compared with CA-125. The model that achieves the highest area under the ROC curve in differentiating benign versus malignant ovarian tumor was ROMA. Cutoff value of HE4 at 70 pMol/L (from 150 pMol/L) would give sensitivity 74.1% and specificity 86.5% that are comparable with ROMA.

**Conclusions:**

HE4 and ROMA had better performance (higher specificity, PPV) compared to CA-125 and RMI. HE4 at 70 pMol/L could be the new cutoff value for Thai women with ovarian cysts, giving higher sensitivity and specificity.

## 1. Introduction

Ovarian cancer is the second most common gynecologic malignancy in Thailand, while the most common cancer is cervical cancer. Ovarian cancer has that highest fatality-to-case ratio and greatest in clinical challenge because they are usually asymptomatic in the early stage. Moreover, the symptoms are vague and unspecific. Five-year survival rate is up to 90% in stage I, up to 70% in stage II, but less than 30% in advanced stage [1]. The earlier stage of diagnosis results in better survival outcome. However, two-thirds of the cases have advanced stage at diagnosis [2]. Moreover, complete tumor resection from primary cytoreductive surgery is one of the most important prognostic factors in ovarian malignancy. The chance of complete cytoreductive surgery depends on stage and operators [2]. There are some research studies to confirm that ovarian cancer patients who were operated by gynecologic oncology surgeons have better survival rate than those operated by general gynecologists [2, 3]. Therefore, there are benefits for the patients, if we can select the cases and send them to the proper operators.

Current diagnostic approaches of ovarian cancer are based on CA-125 and transvaginal ultrasonography and developed the Risk of Malignancy Index (RMI). RMI is an algorithm that employs ultrasound findings and architectural features of a pelvic mass, CA-125 levels, and menopausal status to calculate a numeric score for stratifying patients into high- and low-risk groups for epithelial ovarian cancer (EOC) [4]. However, CA-125 measurement has some limitations. CA-125 does not elevate in half of early-stage EOC patients. On the other hand, it elevates in many benign gynecologic disorders such as endometriosis, PID, benign ovarian tumor, menstruation, pregnancy, or some medical conditions [1, 5]. Therefore, the sensitivity and specificity of CA-125 are not so impressive [6]. For this reason, it is essential to seek for the new marker that has the potential to detect early-stage cancer with high specificity to improve clinical judgement.

HE4 (human epididymis protein 4), a novel biomarker that is expressed in normal glandular epithelium of the female genital tract and breast as well as in a number of glandular carcinomas, has the potential to achieve high sensitivity and specificity for epithelial ovarian cancer detection [7] and also elevated more in early-stage EOC patients compared to CA-125 [8–10].

ROMA (Risk of Ovarian Malignancy Algorithm) is a quantitative test that combines HE4, CA-125, and menopausal status into a numerical score to assess whether a woman who presents with ovarian adnexal mass is at high or low likelihood of finding malignancy on surgery [11, 12].

However, there are some controversies about the use of HE4 that might not be superior to CA-125 in some aspects. Moreover, HE4 does not elevate in some histologic types of EOC [13]. There are just only few studies about HE4 in Asian population especially in Southeast Asian people. Therefore, this study was conducted. Our primary objective was to evaluate the diagnostic value (sensitivity, specificity, negative predictive value, and positive predictive value) of CA-125, RMI, HE4, and ROMA as the diagnostic tools for ovarian cancer discrimination in women with nonfunctional ovarian cysts. Our secondary objective was to evaluate the proper cutoff of HE4 for the population.

## 2. Materials and Methods

This study was a prospective analytic study approved by the Research Ethics Committee of the Faculty of Medicine, Chulalongkorn University, Bangkok, Thailand. Women, aged above than 18 years, with nonfunctional ovarian cysts larger than 3 cm in maximal diameter, scheduled for surgery at King Chulalongkorn Memorial Hospital during 3^rd^ June 2015 and 31^st^ May 2016, were enrolled. Patients with the previous history of ovarian cancer, primary peritoneal cancer or any known malignancies, previous bilateral oophorectomy, pregnancy, or functional cysts were excluded. Written informed consent was obtained from all participants.

Sample size calculation was determined by single population cross-sectional analytic study formula and based upon the proportion of ovarian cancer in overall ovarian masses among women with ovarian mass who underwent surgery at KCMH in the past 10 years (from year 2002 to 2011), that was 13.7% [14]. The sensitivity of HE4 at 80% from the previous study was used for calculation [3, 6]. Drop out rate 20% was added. Therefore, 281 women were required in this study.

Before the operation, 10 ml of blood sample was collected to analyze CA-125 and HE4 by electrochemiluminescence immunoassay (ECLIA) with the same operator and the same machine (MODULAR ANALYTICS E170, cobas e601 and cobas e602 analyzers). Patients' demographic information including age, parity, present of any contraception, and menopausal status were collected. The result of CA-125, RMI, HE4, ROMA, and ultrasound features (size, multiloculation, solid part, bilateral, ascites, and evidence of metastasis) along with pathologic results was also obtained. The ROMA score was calculated automatically by the computer program with a standard formula. The RMI score which included ultrasound imaging score, CA-125 value, and menopause status was calculated by the researcher before the beginning of the operation. The ultrasonogram score was obtained by reviewing the images and official reports of the ultrasound which were performed before admission. During the calculation of the RMI score, the operative finding and pathologic results were unknown. Therefore, the RMI interpreter was blinded to the pathological results to prevent the bias that may occur.

SPSS version 17 (SPSS Inc, Chicago, IL, USA) was used for statistical analysis. General characteristics were analyzed and presented in median, interquartile range, range, and percentage. The diagnostic performances of CA-125, RMI, HE4, and ROMA were evaluated in sensitivity, specificity, NPV, and PPV with the pathologic report which was considered as gold standard. The diagnostic value data were presented in table that would be easy to compare among the tests, and CA-125 was located at the first column which was easy to compare with other tests. The ROC curve of sensitivity and specificity of CA-125, RMI, HE4, and ROMA was plotted to identify the area under the curve of each test. Owing to the cutoff value of ROMA scores that were divided into premenopausal and postmenopausal groups, the ROC curve of premenopausal and postmenopausal groups was plotted. To find the new cutoff value, sensitivity and specificity of each cutoff value of HE4 were generated by computerized program and the most appropriate value was selected and that cutoff value was added into the ROC curve.

## 3. Results

A total of 281 women with nonfunctional ovarian cysts were evaluated. Demographic data are shown in Table 1. Age of the patients ranged from 18 to 79 years old with the mean age of 44 years old (SD ± 13.16 years). Half of the participants were nulliparous. Among this population, 24.2% were postmenopausal group and 19.9% of the final pathological results were malignancy. The level of CA-125, RMI, HE4, and ROMA of all the participants was presented in median, interquartile range, and range, as shown in Table 2.

**Figure 1:**
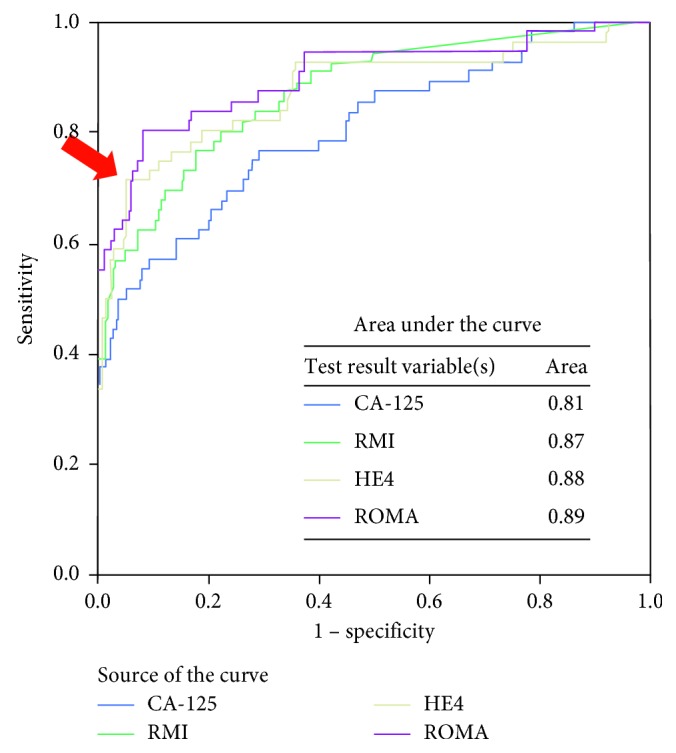
ROC curves and AUC of CA-125, RMI, HE4, and ROMA. The arrow points at the new cutoff point of HE4 at 70 pMol/L have the sensitivity (74.1%) and specificity (86.5%).

**Figure 2:**
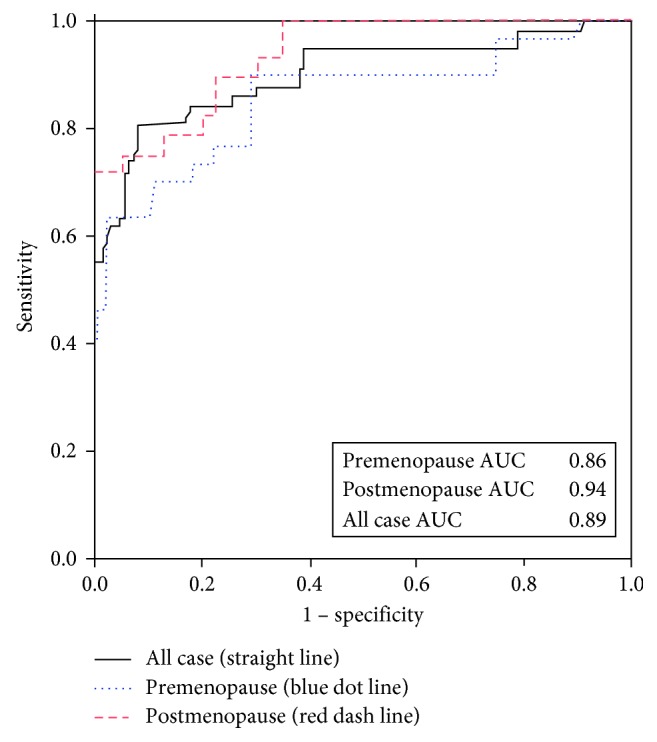
ROC curve of ROMA divided into premenopausal and postmenopausal groups.

Compared to CA-125, HE4 had lower sensitivity (53.4% vs. 87.9%) and NPV (89% vs. 93.6%) but higher specificity (97.8% vs. 46.2%) and PPV (86.1% vs. 29.8%) for differentiating between benign and malignant ovarian tumor. ROMA is slightly lower in sensitivity (79.3% vs. 87.9%) and similar in NPV (93.7% vs. 93.6%), but higher in specificity (79.8% vs. 46.2%) and PPV (50.5% vs. 29.8%) compared to CA-125 (Table 2). ROMA has the highest area under the ROC curve in differentiating benign ovarian cysts versus ovarian cancer (Figure 1). However, the area under the curve among CA-125, RMI, HE4, and ROMA did not have much difference (0.81, 0.87, 0.88, and 0.89, respectively). Focusing on the cutoff level of HE4, when we use the cutoff value of HE4 at 70 pMol/L, it would give the sensitivity (74.1%) and specificity (86.5%) to differentiate between benign and malignant ovarian cancer which were comparable to diagnostic value of ROMA (Table 3). The cutoff value of HE4 at 70 pMol/L was plotted in the ROC curve (Figure 1). Area under the curve of each tests and cutoff are shown in Figure 1. The ROC curve of the HE4 cutoff level at 70 and 140 was plotted to evaluate area under the curve. The AUC of HE4 at the cut point at 70 was 0.87 which was higher than the AUC of the 140 cutoff level (AUC = 0.77). The cut point at 70 of HE4 was plotted in Figure 1. The ROC curve of premenopausal and postmenopausal group of ROMA score was generated and presented as Figure 2. AUC in postmenopausal group of ROMA is higher than that in premenopausal group which was 0.94 vs. 0.86.

## 4. Discussions

This study evaluated the diagnostic performance of CA-125, RMI, HE4, and ROMA in women with nonfunctional ovarian cysts. The diagnostic value includes sensitivity, specificity, NPV, and PPV. Compared to CA-125, HE4 and ROMA had lower sensitivity and NPV, but higher specificity and PPV for differentiating between benign and malignant ovarian tumor. This result was consistent with that of the previous studies by Molina et al. [15], Gorp et al. [16], and Karen et al. [17] that was performed in 6 Asian countries including Thailand.

After generating the ROC, the highest area under the ROC was obtained with ROMA; this denotes that ROMA had the best performance in differentiating benign ovarian cysts versus ovarian cancer with other malignancy which was similar to the previous studies [18, 19]. Moreover, ROMA in postmenopausal group had very high AUC in the ROC curve which was confirmed with the best performance of ROMA in the aspect of diagnostic performance.

Changing the cutoff value of HE4 from 150 pMol/L to 70 pMol/L gives the sensitivity at 74.1% and specificity at 86.5% of HE4 similar to sensitivity and specificity of ROMA. HE4 alone is certainly cheaper than ROMA because ROMA require both CA 125 and HE4. Therefore, HE4 at the cutoff level at 70 pMol/L had comparable diagnostic value with lower cost than ROMA. This point could influence the decision to select diagnostic tools for ovarian cancer prediction. In some situations, such as in primary or secondary care centers, HE4 can be used as the first diagnostic tools for distinguishing pelvic mass before referring the patient to the tertiary care center because there is evidence that ovarian cancer patients who had been performed primary cytoreductive surgery by gynecologic oncology surgeon would have better survival [2]. Therefore, the more accuracy of the predictor we have, the more appropriate management we achieve.

The strength of this study was that there were just only few studies performing in Thailand about HE4 in ovarian cancer detection. Furthermore, we suggest the new cutoff point of HE4 at 70 pMol/L because it gives a comparable sensitivity and specificity to ROMA. Therefore, only HE4 instead of ROMA (HE4 + CA-125) can reduce the cost of investigation. Moreover, this cutoff level of HE4 at 70 pMol/L is consistent with the previous study of Karen et al. [17] that was conducted in Asian population. Therefore, this cutoff level should be more appropriate for Asian and Thai population than the conventional cutoff level at 140 pMol/L.

However, this study still has some limitations. First, this study was performed in single center. This may not include all of the variations in the whole national population. Second, there are only 26 cases of early-stage ovarian cancer that may be too low to evaluate the diagnostic value in this subgroup population. To answer this question, larger trial with specific population should be conducted. Third, the proportion of malignancy in this cohort is 19.9% which was higher than that in normal population because the setting of this study was located in the tertiary care center. Therefore, the diagnostic value may be affected by this factor.

In conclusions, HE4 and ROMA had a better performance to differentiate ovarian cancer from benign ovarian cysts than CA-125 and RMI. The cutoff level 70 pMol/L of HE4 could be the new cutoff values for Asian women with ovarian cysts because it has appropriate sensitivity and specificity to differentiate between malignant and benign ovarian cancer. Moreover, HE4 might be valuable as a first-line biomarker for selecting high risk patients for referral to a tertiary center.

## Figures and Tables

**Table 1:** Patients' characteristics and pathologic results of the ovarian cysts.

Characteristics	Overall *N* (%)
*Age (yr)*	
Mean ± SD	44.13 ± 13.16
Range	18–79

*Parity*	
Nulliparous	165 (58.7%)
Multiparous	116 (41.3%)

*Contraception*	
Yes	24 (8.5%)
No	257 (91.3%)

*Menopausal status*	
Premenopause	213 (75.8%)
Postmenopause	68 (24.2%)

*Pathology*	
Benign	225 (80.1%)
Malignancy	56 (19.9%)
Early-stage ovarian cancer	26 (9.2%)
Advanced-stage ovarian cancer	25 (8.9%)
Other malignancies	5 (1.8%)

**Table 2:** Median, interquartile range, and range and diagnostic value of CA-125, RMI, HE4, and ROMA.

Tools	Median (P25-75)	Range	Sensitivity (%)	Specificity (%)	PPV	NPV
CA-125 (>35 IU/ml)	47.3 (22.1–141.9)	4.77–4,846	87.9	46.2	29.8	93.6
RMI (>250)	35.9 (0–185.5)	0–38,034	64	89.8	61	91
HE4 (>150 pMol/L)	39.7 (20.2–71.6)	27.2–1500	53.4	97.8	86.1	89
ROMA (premenopause)	13.9 (4.5–11.4)	0.9–99.7	66.67	88.52	48.78	94.19
ROMA (postmenopause)	43.9 (10.8–79.1)	2.4–99.4	88.89	70	66.67	90.92

**Table 3:** Compare the sensitivity, specificity, PPV, and NPV of CA-125, HE4, and HE4 at new cutoff point and ROMA.

	CA-125 (>35 IU/mL)	HE4 (>150 pMol/L)	HE4 (>70 pMol/L)	ROMA
Sensitivity (%)	87.9	53.4	74.1	79.3
Specificity (%)	46.2	97.8	86.5	79.8
PPV (%)	29.8	86.1	59.2	50.5
NPV (%)	93.6	89	93.3	93.7
AUC	0.81	0.77	0.87	0.89

## Data Availability

The data used to support the findings of this study are available from the corresponding author upon request.
